# Working memory, mathematics motivation, and problem solving in English learners with and without risk for mathematics learning difficulties: evidence from a high-poverty urban district

**DOI:** 10.3389/fpsyg.2026.1928492

**Published:** 2026-10-19

**Authors:** Michael John Orosco

**Affiliations:** Department of Educational Psychology, University of Kansas, Lawrence, KS, United States

**Keywords:** bilingual learners, elementary mathematics, English learners, mathematics learning difficulties, mathematics motivation, word-problem solving, working memory

## Abstract

This study examined whether working memory (WM) and general mathematics motivation were associated with word-problem-solving accuracy among 76 third-grade Latino English Learners (ELs), 43 of whom were identified as at risk for mathematics learning difficulties (MLD), in a high-poverty urban school district. Students completed WM tasks, a mathematics motivation scale, and standardized word-problem-solving measures. In the primary multilevel model, WM and motivation were each positively and uniquely associated with posttest word-problem-solving accuracy, and both associations remained significant after correction for multiple tests. Both associations also held after controlling for English proficiency and appeared for problems read aloud as well as problems students read independently. However, each association weakened under some alternative analyses. The WM association was no longer significant when prior word-problem performance was controlled, and the motivation association was not significant when motivation was measured at a single time point or when cluster-robust standard errors were used. Exploratory analyses did not detect mediation or moderation, but these tests had low statistical power. The findings offer cautious, correlational evidence that WM and motivation are distinct correlates of word-problem solving among ELs, including those at risk for MLD. Longitudinal and experimental studies are needed to test whether supporting both cognitive and motivational factors improves these students’ outcomes.

## Introduction

English Learners (ELs) make up a substantial share of students in U. S. public schools, yet they continue to perform well below their non-EL peers in mathematics. In fall 2021, ELs accounted for 10.6% of public-school enrollment, or about 5.3 million students (National Center for Education Statistics, 2024). On the 2024 National Assessment of Educational Progress mathematics assessment, Grade 4 ELs again scored well below their non-EL peers, continuing a persistent achievement gap (National Center for Education Statistics, 2025). These disparities are especially consequential for ELs at risk for mathematics learning difficulties (MLD), who must develop a second language while contending with persistent underachievement in mathematics and for whom identifying appropriate educational support is complex.

Word-problem solving is a demanding domain for these students because successful performance requires coordinating linguistic, cognitive, and mathematical processes. Students must interpret problem text in English, map academic mathematical vocabulary (e.g., *altogether*, *difference*, *how many more*) onto developing lexical knowledge, represent the text’s propositional structure, select and execute appropriate arithmetic operations, and maintain relevant information in working memory (WM) throughout the solution process (Orosco et al., 2025; Kintsch and Greeno, 1985; Martiniello, 2009). For ELs at risk for MLD, these linguistic and conceptual processes may impose cumulative cognitive demands that make even grade-appropriate word problems difficult to solve accurately without explicit strategies to reduce text complexity (Orosco and Reed, 2024; Kempert et al., 2011; Swanson et al., 2023). Two sets of individual differences are therefore likely to shape these students’ word-problem performance: the cognitive resources available to manage these demands and the motivation to persist when problems are difficult.

### Working memory and word-problem solving

Baddeley and Hitch’s (1974) multicomponent model conceptualizes WM as a limited-capacity system that temporarily maintains and manipulates information during complex cognition. The original model distinguishes the central executive, which regulates attention, inhibition, and coordination; the phonological loop, which maintains verbal and phonological information; and the visuospatial sketchpad, which maintains and manipulates spatial and pictorial representations. Each component is relevant to mathematical word-problem solving. The central executive supports updating and inhibition; the phonological loop supports rehearsal of verbal problem information; and the visuospatial sketchpad supports the construction of spatial-semantic representations (Swanson and Jerman, 2006; Swanson et al., 2008; see also Peng et al., 2016, for meta-analytic estimates of these associations). In a large sample of 6- to 10-year-olds, all three components predicted word-problem solving; however, reading and calculation skills fully mediated the executive component’s contribution, whereas the storage component retained a direct path to problem-solving accuracy (Fung and Swanson, 2017). Among ELs specifically, longitudinal research indicates that individual differences in WM are robust predictors of mathematical outcomes (Swanson et al., 2018, 2023), and emerging intervention research suggests that WM capacity may predict posttest word-problem accuracy across treatment conditions (Orosco and Reed, 2023, 2024, Orosco et al., 2025).

Baddeley (2000) later added a fourth component, the episodic buffer, a limited-capacity store that binds information from the phonological loop, the visuospatial sketchpad, and long-term memory into integrated, multidimensional episodes (see also Baddeley, 2012). The episodic buffer provides a useful theoretical bridge for understanding ELs’ word-problem solving. To solve a problem presented in a developing second language, a student must temporarily integrate the phonological form of the English text, its propositional meaning, lexical knowledge that may be distributed across Spanish and English, a schematic representation of the problem situation, and stored arithmetic knowledge into a single, coherent problem model (Kintsch and Greeno, 1985). From this perspective, the phonological loop may constrain how much of the English text can be held in mind while the episodic buffer integrates it with conceptual and mathematical knowledge. Although the present battery did not include tasks designed to isolate the episodic buffer, this framework helps explain why complex span tasks (e.g., listening span and conceptual span), which require binding verbal input with semantic knowledge, may be relevant to ELs’ word-problem solving; these integration demands are revisited in the discussion section.

For ELs, phonological WM may be especially salient because word-problem solving requires rapid coordination between printed mathematical language and developing second-language lexical-phonological representations (Martiniello, 2009). When mathematical vocabulary is unstable or underspecified, students may allocate more cognitive resources to maintaining the problem’s language, leaving fewer for mathematical reasoning (Orosco and Reed, 2023, 2024, Orosco et al., 2025; Kempert et al., 2011). Consistent with this account, meta-analytic evidence indicates that the association between WM and second-language reading depends on whether WM is assessed in the first or second language (In’nami et al., 2022), underscoring the relevance of the language in which WM is measured. The phonological-load account is also consistent with meta-analytic evidence that language comprehension, WM, and mathematical vocabulary each uniquely predict word-problem performance (Lin, 2021; see also Peng et al., 2016, for WM specifically). Instructional evidence points in the same direction: Word-problem intervention has improved performance among students experiencing mathematics difficulty (Powell et al., 2022), and findings from the parent intervention study suggest that comprehension strategy instruction, including text-structure analysis, vocabulary instruction, and cognitive chunking, may reduce phonological and WM load for linguistically diverse learners at risk for MLD (Orosco et al., 2025), although this hypothesis awaits independent replication.

### Mathematics motivation and word-problem solving

In this study, general mathematics motivation refers to a cluster of affective-cognitive orientations that includes students’ beliefs about mathematics, perceptions of its value and importance, and behavioral engagement with mathematical tasks (Turner and Meyer, 2009). These elements align with contemporary situated expectancy-value theory, particularly its emphasis on competence beliefs and task value as influences on engagement and persistence (Eccles and Wigfield, 2020). Motivational factors, including effort and persistence, are theorized to contribute to mathematics performance (Eccles and Wigfield, 2020; Middleton and Spanias, 1999; National Mathematics Advisory Panel, 2008), and students who repeatedly struggle with mathematics may develop less positive orientations toward the subject, which may in turn reduce persistence on challenging problem types (Eccles and Wigfield, 2020; Middleton and Spanias, 1999). Prior research indicates that mathematics motivation is associated with achievement, strategy use, help-seeking, and persistence in mathematics learning (Jacobs et al., 2002; Middleton and Spanias, 1999; Ryan and Pintrich, 1997; Wigfield et al., 1997). A recent synthesis likewise underscores the role of motivation and emotion in mathematics learning, suggesting that achievement is shaped not only by cognitive capacity but also by students’ motivational beliefs, emotional experiences, and opportunities for meaningful participation (Schukajlow et al., 2023). This perspective is particularly relevant for ELs and students at risk for MLD, for whom mathematics learning may entail additional linguistic, cognitive, and socioemotional demands. Consistent with this possibility, the relation between mathematics anxiety, a related affective construct, and mathematics competence has been found to differ by mathematics-difficulty status among students (Namkung et al., 2025).

### The present study

Although WM and motivation have each been linked to mathematics performance (Peng et al., 2016; Van der Ven et al., 2023), less is known about how they operate together in word-problem solving among ELs at risk for MLD. Studies that have modeled cognitive and affective predictors jointly have yielded mixed evidence about how the two combine. In a large Dutch sample, mathematics enjoyment predicted story-problem performance alongside WM but did not interact with it (Van der Ven et al., 2023). Among Italian second graders, by contrast, mathematics self-concept moderated the WM–mathematics relation (Passiatore et al., 2024). Other work suggests that affective factors may influence mathematics performance partly through WM (Justicia-Galiano et al., 2017; Pellizzoni et al., 2022). Researchers in this area have noted that few studies examine how cognitive and non-cognitive factors operate together in children’s mathematics learning (Passiatore et al., 2024; Pellizzoni et al., 2022). Moreover, this work has largely used monolingual samples, and some studies explicitly excluded children with identified learning disabilities (e.g., Pellizzoni et al., 2022).

Conversely, research with ELs at risk for mathematics difficulties has focused primarily on WM, language proficiency, and executive processing as predictors of mathematics growth and problem solving (Swanson et al., 2019), with motivational measures rarely included. To our knowledge, no study has examined WM and general mathematics motivation jointly as predictors of word-problem solving among ELs at risk for MLD. It therefore remains unclear whether general mathematics motivation functions as an independent correlate of word-problem solving, overlaps with cognitive resources such as WM, or operates through mediated or moderated pathways. Earlier intervention studies suggest that instructional scaffolds may interact with students’ baseline cognitive and affective profiles to shape problem-solving trajectories (Orosco and Reed, 2023, 2024, Orosco et al., 2025), but this possibility has not been examined directly.

The present study is a secondary analysis of data from an intervention study on comprehension strategy instruction (Orosco et al., 2025) and addresses individual-difference associations rather than intervention effects. Whereas the parent study and related work (Orosco and Reed, 2023, 2024) examined instructional scaffolding and comprehension strategies, the present analysis adds mathematics motivation as a focal predictor alongside WM. Its central contribution is therefore not causal identification but a theory-grounded test of whether cognitive-resource and motivational constructs provide nonredundant information about word-problem performance in an educationally important and understudied population.

Three research questions guided this study. First, is working memory (WM) associated with posttest word-problem-solving accuracy among third-grade English learners (ELs) with and without risk for mathematics learning disability (MLD)? I hypothesized that higher WM would be positively associated with greater posttest word-problem-solving accuracy. Second, when WM and mathematics motivation are entered simultaneously, is each uniquely associated with posttest word-problem-solving accuracy? I hypothesized that both higher WM and greater mathematics motivation would show positive, unique associations with posttest word-problem-solving accuracy after accounting for the other construct. Third, do WM and mathematics motivation show evidence of an indirect or moderated relation to word-problem-solving accuracy? Because Research Question 3 was exploratory, I specified no directional predictions for indirect or moderated relations. Research Questions 1 and 2 constituted the primary hypothesis family, whereas Research Question 3 and all subcomponent, outcome-specific, and sensitivity analyses were considered exploratory. These designations and hypotheses were not preregistered. Because the analyses are secondary and mathematics motivation was measured partly concurrently with the outcome, the term *predictor* is used in the statistical sense and does not imply temporal or causal ordering.

## Method

### Participants

The present study is a secondary analysis of data collected during a completed study of comprehension strategy instruction (Orosco et al., 2025). The analytic sample includes 76 Latino ELs. Unlike the parent study, no exclusion based on fluid reasoning was applied; pretest fluid reasoning was instead retained as a covariate. All data were collected under the parent study’s approved protocol (see the procedure section). Participants were 76 third-grade Latino ELs (40 boys, 36 girls; *M*_age_ = 7.87 years, *SD* = 0.41, range = 7–9 years) from a high-poverty urban public school district in the southwestern United States. Forty-three students were drawn from the parent study’s intervention condition and 33 from its comparison condition. Students were distributed across 12 classrooms. School records indicated that all participants qualified for free or reduced-price school meals.

English proficiency was assessed using the California English Language Development Test (CELDT), the state-administered standardized assessment of English language proficiency. The CELDT assessed listening, speaking, reading, and writing, with internal consistency reliability coefficients ranging from 0.71 to 0.91 across grades and domains; for Grade 3, coefficients ranged from 0.71 to 0.86 (California Department of Education, 2011). Because CELDT scale scores came from the state assessment, item-level data were unavailable, and reliability could not be computed in the current sample. Participants’ proficiency levels ranged from Beginning to Advanced, with most students classified as Early Intermediate or Intermediate, corresponding to developing academic English and emerging sentence- and paragraph-level oral and written production. Spanish was the primary home language for all participants, as documented in the district home-language survey. Instruction across subject areas, including mathematics, was delivered entirely in English, and students also received supplemental English as a second language support during the school day.

Risk status was assigned in the parent study and coincides with condition. Students in the intervention condition were classified as at risk for MLD if (a) school records and teacher reports documented persistent word-problem-solving difficulties across at least three academic years, with no adequate response to general education mathematics instruction, and (b) they performed below the 25th percentile on a standardized word-problem-solving achievement measure at pretest; comparison-condition students were grade-level peers without identified difficulties. Two features of this classification constrain interpretation. First, risk status was defined in the same domain as the outcome, so group differences in posttest accuracy partly reflect the selection criterion; moreover, the cognitive profiles of children classified as having MLD vary with the cutoff used to define the group (Murphy et al., 2007). Second, because risk status and intervention condition coincide by design, their effects cannot be separated. Risk status is therefore not treated as an independent explanatory factor; instead, group differences are described, and exploratory analyses test whether the focal associations differed by group.

### Measures

Because this study is a secondary analysis of Orosco et al., 2025, descriptions of the participants, instruments, and administration procedures may necessarily parallel those of the parent study; the instrument descriptions below are adapted from that source. Table 1 summarizes sample-specific reliability evidence, and Supplementary Table S1 summarizes the content of the motivation items.

**Table 1:** Sample-specific reliability and measurement evidence.

Measure	Index	Estimate	*n*
Working memory			
Nine standardized subtests	Cronbach’s α [95% CI]	0.46 [0.25, 0.64]	66
Nine standardized subtests	McDonald’s ω (one-factor model)	0.46	66
Nine standardized subtests	Subtest intercorrelations, range (median)	−0.23 to 0.35 (0.08)	66–76
Central executive (4 tasks)	Cronbach’s α	0.24	69
Updating task (4 set lengths)	Cronbach’s α	0.79	72
Phonological loop (3 tasks)	Cronbach’s α	0.49	75
Visuospatial sketchpad (2 tasks)	Inter-task r	−0.03	73
Mathematics motivation			
Pretest (10 items)	KR-20 [95% CI]	0.82 [0.73, 0.89]	44
Posttest (10 items)	KR-20 [95% CI]	0.75 [0.66, 0.83]	71
Pretest to posttest	Pearson r	0.18	47
Word-problem solving			
Posttest composite (3 subtests)	Cronbach’s α [95% CI]	0.83 [0.75, 0.89]	72
Pretest composite (3 subtests)	Cronbach’s α [95% CI]	0.67 [0.52, 0.78]	76
CMAT problem solving (24 items)	Pretest–posttest *r*	0.56	72
KeyMath-3 applied problem solving (35 items)	Pretest–posttest *r*	0.64	72
TOMA-2 story problems (25 items)	Pretest–posttest *r*	0.34	72
Covariates			
Raven’s colored progressive matrices (36 items)	Cronbach’s α (3 set scores)	0.51	75
Raven’s colored progressive matrices	Pretest–posttest *r*	0.54	71
TORC-4 text comprehension (60 items)	r with word reading	0.51	73

Estimates were computed in the analytic sample. KR-20 is equivalent to Cronbach’s α for dichotomous items. Pretest–posttest correlations span approximately 10 weeks of intervening instruction and reflect both score stability and intervening change. Item-level data were not available for the working-memory span tasks (other than Updating) or the TORC-4, so internal consistency could not be computed for those measures. CMAT = Comprehensive Mathematical Abilities Test; TOMA-2 = Test of Mathematical Abilities–Second Edition; TORC-4 = Test of Reading Comprehension–Fourth Edition.

#### Working memory

Working memory was assessed with nine individually administered tasks drawn from the S-Cognitive Processing Test (S-CPT; Swanson, 1995) and related procedures (Swanson, 1992, 1996), selected to represent the three components of Baddeley’s model: the central executive (Listening Span, Updating, Conceptual Span, and Digit Sentence Span), the phonological loop (Forward Digit Span, Word Span, and Phonetic Memory Span), and the visuospatial sketchpad (Visual Matrix and Mapping and Directions). Across the complex span tasks, participants held increasingly demanding information in mind while answering related process questions. All WM tasks were administered individually in English by trained examiners following the parent study’s standardized protocol; examiners could clarify task directions in Spanish when needed to ensure comprehension, but all stimuli and responses were in English. Because second-language WM performance is partly constrained by second-language lexical knowledge (In’nami et al., 2022), the language of administration matters when interpreting phonological-loop scores. Reported test–retest reliability for the S-CPT is adequate (*r* = 0.76; Swanson, 1995). In the current sample, internal consistency could be estimated only for the Updating Task, using scores from its four set lengths (Cronbach’s α = 0.79, *n* = 72); for the other eight tasks, the data contained only summary scores rather than trial-level responses, so internal consistency could not be computed. Sample-specific reliability for the nine-task battery is reported in the next paragraph. In prior samples of elementary school children from the same research program, Cronbach’s α exceeded 0.80 for each of the other eight WM tasks (Fung and Swanson, 2017), and KR-20 coefficients were 0.80 for the Updating Task and 0.84 for the Conceptual Span and Digit Sentence Span tasks (Swanson et al., 2015).

##### Working-memory composite and factor structure

Scores on the nine tasks were centered and scaled using fixed constants specified in the analysis code. The standardized scores were then combined into a weighted composite using factor-score weights established in prior research with this battery. These weights were not re-estimated for the present sample. The resulting composite was centered and labeled CFWM. The factor structure was also examined directly in the analytic sample (*n* = 66 with complete data) using maximum likelihood confirmatory factor analysis. The subtests were only weakly intercorrelated (*r*s = −0.23 to 0.35, median *r* = 0.08). A one-factor model fit poorly, χ^2^(27) = 44.09, *p* = 0.020, CFI = 0.52, TLI = 0.36, RMSEA = 0.099, SRMR = 0.094 (Hu and Bentler, 1999), with standardized loadings ranging from −0.24 (Visual Matrix) to 0.54 (Phonetic Memory Span). A correlated three-factor model corresponding to Baddeley’s components produced an inadmissible solution (latent correlations estimated at 1.00) and could not be interpreted. Internal consistency of the nine standardized subtests was low (α = 0.46, 95% CI [0.25, 0.64]; ω = 0.46), as was internal consistency of the component composites (phonological loop, α = 0.49; central executive, α = 0.24); the two visuospatial tasks were uncorrelated (*r* = −0.03). The nine-subtest α is lower than the internal consistency coefficient reported for the WM measures in the parent study (α = 0.84; Orosco et al., 2025); the present value was computed on the nine standardized subtests in the analytic sample used here (*n* = 66) and is therefore the estimate that applies to the scores analyzed in this study. Accordingly, CFWM is interpreted as an aggregate index of performance across diverse WM tasks rather than as a measure of a single latent construct. Low reliability typically attenuates the association between a single predictor and an outcome; with multiple correlated predictors, however, the direction and size of bias depend on the measurement and model assumptions. Because the weighted composite depends on externally derived weights, results are also reported for a unit-weighted composite (the mean of the nine standardized subtests).

##### Listening span

In this task, students heard sets of sentences that gradually increased in number. They were asked to understand each sentence’s meaning and remember the final word. After each set was presented orally, students answered a comprehension question about the content and then recalled the final words of the set. WM capacity was indexed by performance at the largest set size for which students both answered the comprehension question accurately and recalled the target words. The dependent variable was the total number of words recalled correctly, with possible scores ranging from 0 to 8, up to the highest set completed with accurate comprehension responses.

##### Updating task

The Updating Task followed established WM assessment procedures (Swanson, 1995) and used standardized methods to measure executive processing in elementary school children. Students heard sequences of single-digit numbers of varying lengths, with sets containing 9, 7, 5, or 3 digits. Digits were presented at approximately one-second intervals, and no digit appeared more than once within a set. Following each trial, presented in a random set-length order, students recalled the last three numbers in the sequence in the same order they were presented. The dependent variable was the total number of final digits correctly recalled in serial order across trials.

##### Conceptual span task

The Conceptual Span Task measured students’ ability to classify word sequences into higher-order conceptual categories (Swanson, 1992, 1996). Students heard word groups and were instructed to recall words that belonged together. The task increased in difficulty across sets, starting with four words representing two categories in Set 1 and increasing to 20 words representing five categories in Set 7. Scoring reflected the number of correctly recalled words, counted only for sets in which students remembered every item and answered the process question accurately.

##### Digit sentence span task

The Digit Sentence Span Task measured students’ ability to remember numerical information presented in brief sentences (Swanson, 1992, 1996). Students heard digits at approximately one-second intervals and then answered a processing question. Each set contained between 2 and 14 digits. The dependent variable was the total number of digits recalled correctly from sets in which students reproduced all numbers accurately and answered the processing question correctly.

#### Phonological loop tasks

Three storage-only span tasks indexed the phonological loop (Swanson, 1995; Swanson et al., 2008). In Forward Digit Span, students repeated increasingly long strings of digits in order. In Word Span, students recalled sets of unrelated words in serial order. In Phonetic Memory Span, students recalled sets of nonwords in serial order. Each task was scored as the number of items recalled correctly in serial order. Unlike the complex span tasks described above, these tasks did not include a concurrent processing question.

##### Visual–spatial working memory

Two tasks indexed visual–spatial WM: the visual matrix task and the mapping and directions task.

###### Visual matrix task

In the Visual Matrix Task, students studied a matrix dot pattern for roughly 5 s, with embedded process questions verifying that they had attended to the display before recall. Once the pattern was no longer visible, students answered a question about the display and then recreated the dot arrangement on a blank grid. The task grew progressively more difficult, starting with a 2 × 2 grid containing two dots and progressing to an 8 × 8 grid containing 12 dots. Scoring counted correctly reproduced dots, restricted to trials on which the processing question was also answered accurately.

###### Mapping and directions task

The Mapping and Directions Task measured students’ ability to retain a sequence of directions presented on a dot matrix. Students studied the dot map for about 10 s; once it was removed, they answered a question about the display and then recreated the route by linking the dots according to the directional arrows in the original stimulus. Difficulty increased from basic paths with three dots to more complex paths with 12 dots. The outcome measure was the maximum task level at which students correctly reconstructed the path and provided an accurate response to the processing question.

#### Mathematics motivation

Mathematics motivation was assessed with a 10-item scale developed for early elementary students (Orosco, 2016). The scale samples three content areas: beliefs about mathematics (e.g., whether mathematics feels easy or hard), attitudes toward mathematics (e.g., enjoyment and the importance of doing well), and engagement in mathematics (e.g., willingness to spend effort on and choose mathematics activities). Each item presents a pair of statements, one positive and one negative (e.g., “I like doing math” vs. “I do not like doing math”), and students mark the statement that best describes them; positive responses are scored 1 and negative responses 0. The proportion of positive responses yields a score from 0 to 1.00, with higher scores indicating greater motivation. Items were read aloud to students, following the procedure used in the validation study. Item content is summarized in Supplementary Table S1; the full item wording is published in Appendix A of Orosco (2016). In the validation study (Orosco, 2016), conducted with 182 native English-dominant second and third graders, a one-factor model fit well (CFI = 0.987, TLI = 0.987, RMSEA = 0.06), a two-factor model (beliefs/attitudes and engagement) fit statistically equivalently (ΔCFI = 0.008), internal consistency was strong (α = 0.85), one item showed low discrimination, and two items showed differential functioning by gender. Because this evidence comes from a single validation study with a sample that did not include ELs, sample-specific estimates are also reported. In the present sample, internal consistency (KR-20) was 0.82 (95% CI [0.73, 0.89], *n* = 44) at pretest and 0.75 (95% CI [0.66, 0.83], *n* = 71) at posttest. The pretest–posttest correlation was weak, *r*(45) = 0.18, *p* = 0.237, indicating weak rank-order stability of reported motivation across the 10-week study period.

#### Word-problem solving

Word-problem-solving accuracy was assessed using the problem-solving subtests of three individually administered standardized instruments: the Comprehensive Mathematical Abilities Test (CMAT; Hresko et al., 2003), the Test of Mathematical Abilities–Second Edition (TOMA-2; Brown et al., 1994), and the KeyMath-3 Diagnostic Assessment (KeyMath-3; Connolly, 2007). The CMAT Problem Solving, KeyMath-3 Applied Problem Solving, and TOMA-2 Story Problems subtests contain 24, 35, and 25 items, respectively (Orosco et al., 2025; Brown et al., 1994; Connolly, 2007). In a prior sample of second- and third-grade children, KR-20 coefficients were 0.90 for the CMAT, 0.93 for the KeyMath (revised edition), and 0.82 for the TOMA story problems subtest (Swanson et al., 2015). Raw scores were obtained at pretest and posttest, transformed to *z*-scores using fixed constants specified in the analysis code, and a posttest word-problem-solving composite was computed as the mean of the three standardized scores. The composite showed acceptable internal consistency in the current sample (Cronbach’s α = 0.83, *n* = 72) and approximate normality, as indicated by Q–Q plot inspection. A pretest word-problem-solving composite (α = 0.67, *n* = 76) was computed analogously and included as a covariate in a sensitivity analysis. Pretest–posttest correlations, which reflect both score stability and any changes during the intervening instruction, were *r* = 0.56 for the CMAT, 0.64 for the KeyMath-3, and 0.34 for the TOMA-2 (all *n*s = 72). The three subtests differ in their reading demands: CMAT and KeyMath-3 items are read aloud by the examiner, whereas the TOMA-2 requires students to read problems independently under a time limit. Because a composite that combines these formats could conflate problem solving with independent English reading, the primary analyses were repeated separately for the orally administered subtests (the mean of the standardized CMAT and KeyMath-3 scores) and for the TOMA-2.

##### Applied problem solving

Students completed the Applied Problem-Solving subtest (Applications domain) of the KeyMath-3 Diagnostic Assessment (Connolly, 2007) in individual sessions; this subtest measures children’s ability to solve word problems presented orally by the examiner. The subtest includes items that increase in difficulty and require students to interpret orally presented problem situations and determine the appropriate mathematical operations or procedures. Administration ended after students provided four consecutive incorrect responses.

##### Oral word-problem solving

Students’ word-problem-solving skills were measured using the Problem-Solving subtest of the Comprehensive Mathematical Abilities Test (CMAT-PS; Hresko et al., 2003), administered individually. This subtest evaluates students’ ability to understand and solve orally presented word problems. Items are ordered by increasing difficulty and assess problem-solving using the four basic arithmetic operations—addition, subtraction, multiplication, and division—as well as multistep problems that require multiple operations. Consistent with standardized administration procedures, trained examiners read each item aloud and repeated it once, as needed. Students could respond either verbally or in writing, and they were provided with paper and pencil to complete calculations. The task was not timed, allowing assessment of mathematical problem-solving skills without added processing-speed demands. Administration was discontinued after three consecutive incorrect responses.

##### Reading word-problem solving

The Story Problems subtest of the Test of Mathematical Abilities–Second Edition (TOMA-2; Brown et al., 1994) was administered under standardized procedures to small groups of five to eight children. This subtest consists of written word problems that progressively increase in complexity and require students to read and solve each item independently within a 10-min time limit. Items range from basic one-step problems to multistep problems that require the application and integration of multiple mathematical concepts. Students entered their responses directly in the test booklets and provided written work when appropriate. To minimize frustration, the administration ended after three consecutive incorrect responses.

#### Reading comprehension

Silent Reading comprehension was measured using the text comprehension subtest of the test of reading comprehension–fourth edition (TORC-4; Brown et al., 2009), which contains 12 passages with five multiple-choice questions each (60 items). Students independently read short passages and answered multiple-choice questions about each passage. Raw scores represented the total number of correct responses and were standardized within the study sample for the analyses. The text comprehension subtest demonstrates strong reliability across age groups and subgroups in the normative sample, including evidence of high internal consistency and test stability (Brown et al., 2009). Item-level data were not available, and the TORC-4 was administered only at pretest; therefore, neither internal consistency nor a pretest–posttest correlation could be computed in the current sample. In the present sample, TORC-4 raw scores were also moderately correlated with a standardized measure of word reading administered at pretest, r(71) = 0.51, *p* < 0.001, providing evidence of convergent validity.

#### Fluid reasoning

Fluid reasoning was assessed with Raven’s colored progressive matrices (Raven et al., 1990; raw score range = 11–33, *M* = 22.65, *SD* = 5.02). The test contains 36 items in three sets of 12. In the current sample, internal consistency across the three set scores was Cronbach’s α = 0.51 (*n* = 75), and the pretest–posttest correlation was *r* = 0.54 (*n* = 71). Because the α estimate is based on only three set scores that differ substantially in difficulty, it likely underestimates item-level internal consistency.

### Procedure

The parent study’s research protocol was approved by the institutional review board of the authors’ institution, and participants’ parents or legal guardians provided written informed consent; the present secondary analyses were conducted under that approved protocol. All participants received general education mathematics instruction using a direct instructional sequence that primarily emphasized procedural skill development; students in the intervention condition also received the comprehension strategy instruction described in Orosco et al. (2025). Trained examiners administered pretest assessments of WM, mathematics motivation, fluid reasoning, reading comprehension, and word-problem-solving accuracy individually in approximately 30-min sessions, except the TOMA-2, which was administered in small groups. All cognitive and achievement measures were administered in English, consistent with the language of mathematics instruction in participating classrooms. Posttest assessments of word-problem solving and motivation were administered approximately 10 weeks later. The intervention condition was not a predictor in the present analyses but was included in a sensitivity analysis.

### Analytic strategy

All statistical analyses were performed using SAS version 9.4 (SAS Institute Inc., 2013). Multilevel models were estimated with PROC MIXED using restricted maximum likelihood (REML). Multilevel modeling was selected because students were nested within 12 classrooms (Raudenbush and Bryk, 2002). The unconditional model yielded an intraclass correlation coefficient of essentially zero, indicating negligible systematic between-classroom variance in posttest word-problem-solving accuracy. Multilevel models were retained to reflect the nested design while allowing for classroom clustering, but classroom-level variance components were not substantively interpreted. To permit stable estimation given the boundary-zero classroom variance, PROC MIXED was run with the NOBOUND option. As a complementary sensitivity analysis, single-level ordinary least squares models with classroom-clustered robust standard errors were estimated in SAS using PROC SURVEYREG, with classroom specified as the clustering unit (Cameron and Miller, 2015). The −2 log likelihood, Akaike information criterion (AIC), and Bayesian information criterion (BIC) are reported for descriptive purposes; because the models were estimated by REML, differ in their fixed effects, and were fit to different numbers of cases, these statistics were not used for formal model comparison. Confirmatory factor analyses, multiple imputation, multiplicity adjustments, and Monte Carlo power analyses were conducted after the primary estimates had been reproduced from the same data files.

Three sequential models addressed the first two research questions. Model 1 partitioned total posttest word-problem-solving variance into between- and within-classroom components. Model 2 added the centered full WM composite (CFWM) as the sole predictor. Model 3, the joint model, added standardized mathematics motivation to CFWM. Model 3 was designated the primary model because it tested whether the two theoretically central constructs showed unique associations with word-problem-solving accuracy without introducing covariates that could overcontrol shared construct-relevant variance. Specifically, reading comprehension and WM are moderately correlated (Peng et al., 2018) and theoretically intertwined during second-language mathematical text processing; therefore, controlling for reading comprehension when testing WM may partial out variance that is constitutive of the cognitive process rather than extraneous to it. For transparency, Model 4 included standardized pretest fluid reasoning and standardized reading comprehension as covariates in a robustness check. Finally, in an exploratory model, CFWM was replaced with standardized composites of the central executive, phonological loop, and visuospatial sketchpad. Because these composites were correlated and had low reliability, a Wald test evaluated whether the phonological-loop and central-executive coefficients differed, rather than inferring dissociation from separate significance tests.

The third research question was addressed through exploratory analyses of indirect effects and interactions. Pretest and posttest motivation scores were averaged to form a single composite. Averaging across time points was intended to reduce occasion-specific variation (Rushton et al., 1983) and to retain students with only one administration (pretest motivation was missing for 28 students). However, the pre–post correlation was weak (*r* = 0.18), so the composite is best interpreted as students’ average reported motivation across the study period rather than as a stable trait; pretest-only and posttest-only analyses are reported as sensitivity checks. Because the composite combined measurements obtained before and after the intervention, temporal precedence of the mediator could not be established; the indirect-effect model was therefore interpreted as a test of statistical rather than causal mediation (Bullock and Green, 2021; Maxwell and Cole, 2007; Rohrer et al., 2022). Path *a* regressed standardized motivation on CFWM, with gender as a covariate. Paths *b* and *c*′ regressed posttest word-problem-solving accuracy on motivation and CFWM, respectively, controlling for gender. The indirect effect (*a* × *b*) and its 95% confidence interval were estimated using a Monte Carlo procedure in SAS with 10,000 simulated draws from the joint sampling distribution of *a* and *b* (MacKinnon et al., 2004; Pesigan and Cheung, 2024; Preacher and Selig, 2012; Tofighi and MacKinnon, 2016). A CFWM × Motivation interaction was tested as a moderator. Monte Carlo simulations (2,000 replications for the interaction; 1,000 for the indirect effect, each with 2,000 Monte Carlo draws) estimated the power of these tests at *n* = 62, using the observed Model 3 coefficients as population values for the remaining paths (Fritz and MacKinnon, 2007; McClelland and Judd, 1993).

Sensitivity analyses assessed the stability of the findings by estimating (a) the covariate-adjusted Model 4; (b) Model 3 with the intervention condition added; (c) Model 3 with pretest word-problem-solving accuracy added; (d) Model 3 with pretest-only, and separately posttest-only, motivation replacing the averaged composite; (e) Model 4 as a single-level ordinary least squares regression with classroom-clustered robust standard errors; (f) Model 3 with English language proficiency added; (g) Models 3 and 4 with the unit-weighted WM composite; (h) Model 3 estimated separately for the orally administered composite and for the TOMA-2; and (i) Model 3 with risk status and its interactions with WM and motivation. All focal estimates are summarized in Supplementary Table S2.

#### Missing data

Missing observations were concentrated in the predictors (CFWM, *n* = 10; motivation, *n* = 3; reading comprehension, *n* = 2; pretest-only motivation, *n* = 28) and the posttest outcome (*n* = 4). The primary and sensitivity models were estimated with complete-case (listwise) deletion, the PROC MIXED default, so sample sizes varied across models. Students missing CFWM did not differ significantly from other students on posttest word-problem accuracy, motivation, reading comprehension, or fluid reasoning (all *p*s > 0.07), although their pretest word-problem scores were somewhat higher (*M* = 0.13 vs. −0.34, *p* = 0.074). To evaluate the robustness of the complete-case estimates under the less restrictive missing-at-random assumption, Model 3, Model 4, the pretest-adjusted model, and the subcomponent model were re-estimated after multiple imputation by chained equations (*m* = 50; van Buuren, 2018). The imputation model included all analysis variables plus auxiliary variables (pretest word-problem accuracy, English language proficiency, risk status, gender, age, the WM component composites, pretest and posttest motivation, and the oral and TOMA-2 outcome scores). Estimates were pooled with Rubin’s (1987) rules using Barnard and Rubin’s (1999) small-sample degrees of freedom. Because the classroom variance was estimated at zero, the imputed-data models were estimated as single-level regressions.

#### Adjustment for multiple testing

Two families of tests were distinguished. The confirmatory family comprised the three tests corresponding to Research Questions 1 and 2 (the CFWM coefficient in Model 2 and the CFWM and motivation coefficients in Model 3), to which the Holm (1979) sequential correction was applied. The exploratory family comprised the 34 focal coefficient tests from the subcomponent, indirect-effect, interaction, and sensitivity models, for which false-discovery-rate *q* values from the Benjamini and Hochberg (1995) procedure are reported alongside unadjusted *p* values (Supplementary Table S2). Exploratory results are interpreted as hypothesis-generating regardless of their unadjusted significance.

The analytic strategy prioritized transparency over parsimony. The primary model tested the theoretically central cognitive and motivational associations, while sensitivity models evaluated how those associations changed under alternative assumptions about prior achievement, broader cognitive covariates, intervention exposure, and classroom clustering. This approach is appropriate for a secondary analysis because it makes specification dependence visible rather than treating a single model as definitive.

## Results

### Preliminary analyses

Descriptive statistics and bivariate correlations are presented in Table 2. A general linear model indicated that gender was not significantly associated with posttest word-problem-solving accuracy, *F*(1, 70) = 0.77, *p* = 0.383. Reliability estimates and the WM factor-structure results are reported in the measures section and Table 1.

**Table 2:** Descriptive statistics and Pearson correlations among major study variables.

Variable	N	M	SD	1	2	3	4	5	6
1. WPS composite, posttest (z)	72	−0.12	0.81	—					
2. WPS composite, pretest (z)	76	−0.28	0.65	0.70***	—				
3. Working memory (CFWM)	66	−0.04	0.97	0.41**	0.45***	—			
4. Mathematics motivation	73	0.78	0.22	0.31**	0.19	0.12	—		
5. Fluid reasoning (Raven, z)	75	0.00	1.00	0.29*	0.23	0.13	0.08	—	
6. Reading comprehension (TORC, z)	74	0.00	1.00	0.44***	0.36**	0.44***	0.32**	0.24*	—

Correlations are Pearson *r* values computed pairwise on available cases. WPS Composite (Pretest and Posttest) = mean of standardized CMAT, KeyMath-3, and TOMA-2 problem-solving raw scores; the *z*-score transformations used fixed centering and scaling constants specified in the analysis code. CFWM = centered full working-memory composite, derived as a weighted linear combination of nine standardized working-memory subtests using previously estimated factor-score weights (see Measures for factor-structure results in the present sample). Mathematics Motivation = mathematics motivation measure endorsement proportion averaged across pretest and posttest (range 0–1.00). TORC = Test of Reading Comprehension–Fourth Edition. *p < 0.05. **p < 0.01. ***p < 0.001.

The three posttest word-problem-solving measures were strongly intercorrelated (*r*s = 0.55–0.70, all *p*s < 0.001), supporting the use of the composite outcome. CFWM was significantly correlated with the standardized word-problem-solving composite, *r*(60) = 0.41, *p* = 0.001. Mathematics motivation was also associated with the standardized word-problem-solving composite, *r*(70) = 0.31, *p* = 0.009. WM and mathematics motivation were not significantly correlated, *r*(61) = 0.12, *p* = 0.348, consistent with limited overlap between their observed scores in this sample.

### Multilevel regression models

To address the first two research questions, a sequence of hierarchical multilevel models was estimated to predict posttest word-problem-solving accuracy. Model 1 was the unconditional model, which partitioned outcome variance into between- and within-classroom components. Model 2 added the centered full WM composite (CFWM) as the sole predictor, and Model 3, the primary joint model, added standardized mathematics motivation to CFWM. Model 4, a covariate-adjusted robustness check, is reported in the Sensitivity Analyses section. Table 3 presents fixed effects, variance components, and fit statistics for all models, and inferences are based on fixed-effect tests rather than comparisons of REML fit statistics across models estimated on different cases.

**Table 3:** Hierarchical multilevel models predicting posttest word-problem-solving accuracy.

Parameter	Model 1	Model 2	Model 3	Model 4
Fixed effects				
Intercept	−0.13 (0.07)	−0.10 (0.05)	−0.12 (0.07)	−0.16 (0.09)
CFWM	—	0.33 (0.09)***	0.28 (0.09)**	0.18 (0.10)
Mathematics motivation	—	—	0.22 (0.09)*	0.20 (0.10)*
Fluid reasoning	—	—	—	0.12 (0.10)
Reading comprehension	—	—	—	0.17 (0.10)
Random effects				
Residual variance	0.69 (0.12)***	0.54 (0.10)***	0.48 (0.09)***	0.45 (0.08)***
Fit statistics				
−2 log likelihood	176.1	137.1	134.6	131.4
AIC	180.1	141.1	138.6	135.4
BIC	181.1	142.0	139.4	136.4
N	72	62	62	60

Cell entries are unstandardized fixed-effect coefficients (SE). Model 3 is the joint test of working memory and mathematics motivation as simultaneous correlates of posttest word-problem-solving accuracy. Model 4 is a covariate-adjusted robustness check; see Results and Discussion for interpretation. All predictors except CFWM (which is centered) were transformed using fixed centering and scaling constants specified in the analysis code. CFWM = centered full working-memory composite; mathematics motivation = standardized mathematics motivation score, averaged across pretest and posttest; fluid reasoning = standardized pretest Raven’s Colored Progressive Matrices score; reading comprehension = standardized Test of Reading Comprehension–Fourth Edition (TORC-4) score. Models were estimated by REML with the NOBOUND option (SAS 9.4 PROC MIXED). Sample sizes vary across models because of missing data in the predictors (complete-case estimation). Because the models were estimated by REML on different numbers of cases, fit statistics are descriptive and are not comparable across models. For the three confirmatory tests (CFWM in Models 2 and 3; motivation in Model 3), Holm-adjusted *p*-values were 0.002, 0.008, and 0.019, respectively. *p < 0.05. **p < 0.01. ***p < 0.001.

#### Unconditional model (model 1)

The unconditional model contained no predictors and served as the baseline for partitioning outcome variance. The fixed intercept was −0.13 (*SE* = 0.07), and the residual (within-classroom) variance was 0.69 (*SE* = 0.12, *p* < 0.001). Consistent with the boundary-zero classroom variance reported in the Analytic Strategy, the intraclass correlation coefficient was essentially zero, indicating that virtually all variance in posttest word-problem-solving accuracy was within classrooms rather than between them. Because between-classroom variance was negligible, classroom-level variance components were not substantively interpreted; the multilevel specification was nonetheless retained in subsequent models to reflect the nested design. Fit statistics for Model 1 were −2 log likelihood = 176.1, AIC = 180.1, and BIC = 181.1 (*N* = 72).

#### Working memory model (model 2)

WM was significantly associated with posttest word-problem-solving accuracy, *b* = 0.33, *SE* = 0.09, 95% CI [0.14, 0.51], *t*(50) = 3.57, *p* < 0.001, and the association remained significant after Holm correction (adjusted *p* = 0.002). Because Models 1 and 2 were estimated on different numbers of cases (72 vs. 62), their fit statistics and residual variances are not directly comparable. In the 62 cases included in Model 2, WM accounted for approximately 16% of the variance in posttest word-problem-solving accuracy (*R*^2^ = 0.16). This result provided an affirmative answer to Research Question 1.

#### Joint model (model 3)

Model 3 added standardized mathematics motivation to CFWM. When estimated simultaneously, both predictors were significantly associated with posttest word-problem-solving accuracy. WM remained significant, *b* = 0.28, *SE* = 0.09, 95% CI [0.11, 0.47], *t*(49) = 3.05, *p* = 0.004. Mathematics motivation was also significant, *b* = 0.22, *SE* = 0.09, 95% CI [0.05, 0.42], *t*(49) = 2.43, *p* = 0.019. Both coefficients remained significant after Holm correction for the three confirmatory tests (adjusted *p*s = 0.008 for WM and 0.019 for motivation). In the same 62 cases, adding motivation increased the proportion of explained variance from 0.16 to 0.25, and residual variance decreased from 0.54 to 0.48, consistent with motivation providing additional explanatory information in this sample. These results provided an affirmative answer to Research Question 2 in the multilevel specification.

#### Exploratory working-memory subcomponents

In the exploratory subcomponent model (Supplementary Table S2), the phonological loop was the only WM component that reached nominal statistical significance, *b* = 0.29, *SE* = 0.13, *t*(57) = 2.32, *p* = 0.024. The central-executive coefficient was numerically larger but not significant, *b* = 0.36, *SE* = 0.18, *t*(57) = 1.94, *p* = 0.058, and the visuospatial sketchpad was not significant, *b* = −0.02, *SE* = 0.12, *t*(57) = −0.18, *p* = 0.854. Several results indicate that this pattern does not constitute evidence of a dissociation. In a re-estimation with all available cases (*n* = 72), the phonological-loop and central-executive coefficients did not differ, difference = −0.04, *SE* = 0.26, *t*(68) = −0.15, *p* = 0.884. The phonological-loop coefficient did not survive false-discovery-rate correction (*q* = 0.054), and neither component was significant when motivation, reading comprehension, and fluid reasoning were added (phonological loop, *b* = 0.20, *p* = 0.152; central executive, *b* = 0.25, *p* = 0.175; *n* = 69). The pattern is therefore treated as hypothesis-generating, particularly given the low reliability of the component composites and the lack of preregistration.

#### Indirect-effect and interaction analyses

The exploratory indirect-effect analysis did not detect an indirect association through motivation. Path *a*, regressing standardized motivation on CFWM with gender as a covariate, was not significant, *b* = 0.09, *SE* = 0.14, *t*(60) = 0.62, *p* = 0.533. In the joint multilevel model estimating Paths *b* and *c′*, both predictors showed significant direct associations with posttest word-problem-solving accuracy: Path *b* for motivation, *b* = 0.25, *SE* = 0.10, *p* = 0.013, and Path *c′* for WM, *b* = 0.30, *SE* = 0.10, *p* = 0.002. The indirect effect was near zero (*ab* = 0.02), and the Monte Carlo 95% CI [−0.05, 0.10] included zero. The formal CFWM × Motivation interaction was also nonsignificant, *b* = 0.03, *SE* = 0.10, *p* = 0.803. These results did not support mediation or moderation, but they should not be read as evidence that such effects are absent. Simulation-based power at *n* = 62 was 0.38 to detect an interaction coefficient of 0.20 and reached 0.80 only for a coefficient of about 0.35 (*f*^2^ ≈ 0.14). Power to detect the indirect effect was 0.10, 0.26, and 0.43 when the WM-to-motivation path was small (*a* = 0.14), intermediate (*a* = 0.26), or medium (*a* = 0.39), respectively. The observed confidence intervals (indirect effect, [−0.05, 0.10]; interaction, [−0.17, 0.22]) are compatible with no effect but also with small-to-moderate effects; the mediation and moderation tests are therefore inconclusive.

### Sensitivity analyses

#### Covariate-adjusted model

When standardized pretest measures of fluid reasoning and reading comprehension were added to Model 3, the WM coefficient was attenuated and no longer statistically significant at the conventional level (*b* = 0.18, *SE* = 0.10, 95% CI [−0.02, 0.38], *p* = 0.071). Mathematics motivation remained significant, *b* = 0.20, *SE* = 0.10, 95% CI [0.01, 0.39], *p* = 0.040. Pretest fluid reasoning was not significant, *b* = 0.12, *SE* = 0.10, *p* = 0.243, and reading comprehension was not significant, *b* = 0.17, *SE* = 0.10, *p* = 0.110. The attenuation of the WM coefficient is consistent with the correlation between WM and reading comprehension (*r*[63] = 0.44, *p* < 0.001).

#### Pretest word-problem-solving accuracy

When pretest word-problem-solving accuracy was added to Model 3 as an autoregressive covariate, pretest performance was strongly associated with posttest performance, *b* = 0.69, *SE* = 0.15, *p* < 0.001. Mathematics motivation met the unadjusted 0.05 threshold, *b* = 0.16, *SE* = 0.08, *p* = 0.047, whereas WM was no longer significant, *b* = 0.11, *SE* = 0.09, *p* = 0.211. This pattern is expected when a prior-outcome covariate accounts for substantial stable individual-difference variance.

#### Single-administration motivation scores

When pretest-only motivation replaced the averaged motivation composite, the analytic sample was reduced to *n* = 40 due to missing pretest motivation data. Pretest-only motivation was not significantly associated with posttest word-problem-solving accuracy, *b* = 0.22, *SE* = 0.19, *p* = 0.234, whereas the WM coefficient was not significant, *b* = 0.24, *SE* = 0.13, *p* = 0.059. When posttest-only motivation was used (*n* = 62), the motivation coefficient was positive but not significant, *b* = 0.16, *SE* = 0.09, *p* = 0.060, whereas WM remained significant, *b* = 0.32, *SE* = 0.09, *p* < 0.001. Together, these analyses indicate that evidence for the motivation association depends on the choice of administration and should be interpreted as contemporaneous rather than prospective.

#### Single-level model with cluster-robust standard errors

Given the near-zero intraclass correlation, Model 4 was also estimated as a single-level ordinary least squares regression with classroom-clustered robust standard errors. In this specification, WM was significant (*b* = 0.18, robust *SE* = 0.09, 95% CI [0.02, 0.35], *p* = 0.032), whereas mathematics motivation was not (*b* = 0.20, robust *SE* = 0.14, 95% CI [−0.07, 0.47], *p* = 0.143). Because cluster-robust inference can be unstable with few clusters, interpret this analysis as a robustness check rather than a definitive alternative model. Across specifications, evidence for WM was strongest in models without reading comprehension as a covariate and in the cluster-robust specification, whereas evidence for motivation was strongest in multilevel specifications.

#### English language proficiency

English language proficiency was examined as a covariate. Model 3 was re-estimated with standardized state English language development scale scores added (*n* = 57). English proficiency was independently associated with posttest accuracy, *b* = 0.26, *SE* = 0.09, *p* = 0.004, and both WM, *b* = 0.23, *SE* = 0.10, *p* = 0.026, and motivation, *b* = 0.25, *SE* = 0.09, *p* = 0.006, remained significant. English proficiency was weakly correlated with WM (*r* = 0.12) and uncorrelated with motivation (*r* = −0.05).

#### Multiple imputation

With all 76 students retained through multiple imputation, the Model 3 estimates were similar to the complete-case estimates: WM, *b* = 0.30, *SE* = 0.08, 95% CI [0.13, 0.47], *p* < 0.001; motivation, *b* = 0.20, *SE* = 0.08, 95% CI [0.03, 0.36], *p* = 0.022. In the covariate-adjusted Model 4, the pattern differed from the complete-case analysis: WM was significant, *b* = 0.23, *SE* = 0.09, *p* = 0.011, whereas motivation was not, *b* = 0.14, *SE* = 0.08, *p* = 0.105. With pretest word-problem accuracy controlled, neither WM (*b* = 0.09, *p* = 0.218) nor motivation (*b* = 0.12, *p* = 0.071) was significant. Fractions of missing information were 0.15 or lower for all focal coefficients.

#### Unit-weighted working-memory composite

Replacing CFWM with the unit-weighted composite (*n* = 72) yielded results similar to Model 3 (WM, *b* = 0.31, *SE* = 0.09, *p* < 0.001; motivation, *b* = 0.24, *SE* = 0.09, *p* = 0.009). In the covariate-adjusted analogue of Model 4 (*n* = 69), WM remained significant, *b* = 0.23, *SE* = 0.09, *p* = 0.007, whereas motivation did not, *b* = 0.17, *SE* = 0.09, *p* = 0.070. Thus, the attenuation of the WM coefficient in the complete-case Model 4 did not recur with the unit-weighted composite or with multiple imputation.

#### Oral versus Reading-dependent outcomes

WM and motivation were each associated with both the orally administered composite (WM, *b* = 0.31, *SE* = 0.10, *p* = 0.003; motivation, *b* = 0.24, *SE* = 0.10, *p* = 0.023) and the reading-dependent TOMA-2 (WM, *b* = 0.24, *SE* = 0.10, *p* = 0.019; motivation, *b* = 0.24, *SE* = 0.10, *p* = 0.018). Thus, the focal associations were not driven solely by the subtest that required independent English reading. In the subcomponent model, the phonological-loop coefficient was nominally significant for the oral composite (*b* = 0.35, *p* = 0.015) but not for the TOMA-2 (*b* = 0.22, *p* = 0.110); this pattern is consistent with the demand of holding orally presented problem text in mind, but it should be interpreted cautiously given the number of tests.

#### Multiplicity-adjusted summary

Supplementary Table S2 reports every focal test with its unadjusted and adjusted values. The three confirmatory tests remained significant after Holm correction. Of the 34 exploratory and sensitivity tests, 19 were nominally significant, and 10 of these had *q* < 0.05 (Supplementary Table S2): seven WM coefficients and three motivation coefficients. The remaining nominally significant results, including the phonological-loop coefficient, had *q* values between 0.054 and 0.084. Overall, evidence for WM was somewhat more robust to multiplicity adjustment and alternative specifications than evidence for motivation.

## Discussion

The primary objective of this study was to determine whether WM and general mathematics motivation provide distinct, nonredundant information about word-problem-solving accuracy among third-grade Latino ELs with and without MLD risk. In the primary multilevel model, both constructs were positively associated with posttest word-problem accuracy when entered simultaneously, and both associations survived correction for the three confirmatory tests. However, these associations were specification-dependent. The WM coefficient was attenuated when reading comprehension and fluid reasoning were controlled in the complete-case analysis (although not with multiple imputation or the unit-weighted composite) and when pretest word-problem accuracy was controlled. The motivation coefficient was attenuated under cluster-robust inference, when a single motivation administration was used, in the multiply imputed models controlling for pretest word-problem accuracy or for cognitive covariates, and in the covariate-adjusted model with the unit-weighted WM composite. Both associations were robust to adjustment for English language proficiency and were observed for both orally administered and reading-dependent outcomes. Exploratory tests did not detect mediation or moderation, but these tests had limited power. The findings are therefore interpreted as cautious, correlational evidence that WM and motivation are distinct correlates of word-problem performance in this sample, not as evidence of causal or independent pathways.

### Working memory and word-problem solving

Consistent with prior WM and mathematics research (Orosco and Reed, 2024; Fung and Swanson, 2017; Swanson and Jerman, 2006; Swanson et al., 2008), WM was associated with posttest word-problem-solving accuracy and, when entered alone, accounted for approximately 16% of the variance in posttest accuracy. The association remained significant when mathematics motivation was added, suggesting that WM captured variance in word-problem performance that was not shared with students’ motivational orientation.

The WM coefficient was attenuated when reading comprehension and fluid reasoning were included, but this attenuation should be interpreted neither as evidence that WM is irrelevant nor as evidence that the two constructs are inseparable. Reading comprehension and WM were moderately correlated in this sample (*r* = 0.44), consistent with meta-analytic evidence of a moderate association between WM and reading (Peng et al., 2018) and with evidence that reading and calculation skills mediate much of the executive component’s contribution to word-problem solving (Fung and Swanson, 2017). With 60 cases and correlated predictors, the covariate-adjusted model had limited precision for apportioning shared variance, and the attenuation did not recur when missing data were multiply imputed or when the unit-weighted composite was used. Similarly, the loss of significance when pretest word-problem accuracy was controlled is expected when a prior measure of the outcome absorbs stable individual differences, including those associated with WM. Separating the contributions of WM and reading comprehension would require designs that measure both repeatedly and that experimentally vary the reading demands of problems (Lin, 2021; Powell et al., 2022).

### Exploratory working-memory subcomponent pattern

In the exploratory subcomponent model, the phonological-loop coefficient reached nominal significance, whereas the central-executive coefficient, which was numerically larger, did not. Because the two coefficients did not differ, the phonological-loop result did not survive false-discovery-rate correction, neither component was significant after covariate adjustment, and the component composites had low internal consistency, these results do not establish that phonological WM is more important than executive WM for ELs. The pattern is reported because it bears on a theoretically motivated hypothesis: When learners solve linguistically dense problems in a developing second language, WM demands may shift toward maintaining phonological representations of the problem text (Martiniello, 2009), with the episodic buffer integrating those representations with conceptual and arithmetic knowledge (Baddeley, 2000, 2012). This hypothesis is consistent with evidence that the storage component of WM retains a direct path to word-problem solving after reading and calculation are controlled (Fung and Swanson, 2017). It contrasts, however, with meta-analytic evidence in which the central executive shows the strongest association with mathematical problem solving (Ji and Guo, 2023; Peng et al., 2016). This interpretation is speculative; testing it will require larger, preregistered studies with reliable component measures administered in both of students’ languages.

### Mathematics motivation and word-problem solving

Mathematics motivation, operationalized as general mathematics beliefs, attitudes, and engagement on the mathematics motivation measure, was positively associated with posttest word-problem-solving accuracy in the multilevel model. This result aligns with expectancy-value and engagement frameworks that conceptualize motivation as a contributor to learning outcomes distinct from cognitive resources (Eccles and Wigfield, 2020; National Mathematics Advisory Panel, 2008; Schukajlow et al., 2023). In correlational terms, students who reported more positive mathematics beliefs, value, and engagement also tended to have higher posttest word-problem accuracy. The design does not permit inferences about whether motivation contributed to accuracy, accuracy contributed to motivation, or both reflected other factors.

This conclusion warrants caution. The motivation score was the average of the pretest and posttest administrations of the mathematics motivation measure, the pretest–posttest correlation of the measure was weak (*r* = 0.18), and neither the pretest-only analysis, which was underpowered, nor the posttest-only analysis yielded a significant coefficient. Therefore, the evidence supports motivation as a contemporaneous correlate of posttest word-problem-solving accuracy rather than as a clearly antecedent causal predictor. The study also did not measure implicit theories of intelligence; therefore, the findings should not be interpreted as evidence for growth-mindset interventions (Dweck, 2006).

### Distinct correlates rather than established pathways

The indirect-effect model did not show that motivation statistically mediated the association between WM and word-problem solving, the interaction model did not show that motivation moderated that association, and WM and motivation were only weakly correlated. These results are consistent with WM and motivation contributing additively to word-problem performance, but they do not establish that pattern. The simulation-based power of the indirect-effect and interaction tests was low for small-to-moderate effects, and the confidence intervals include educationally meaningful effects. Moreover, because the motivation composite combined pretest and posttest administrations, the temporal ordering required for mediation could not be established (Maxwell and Cole, 2007). WM and motivation are therefore described as distinct correlates rather than as parallel causal pathways. If adequately powered longitudinal studies confirm an additive pattern, interventions that target only cognitive load or only engagement might leave part of the relevant variance unaddressed; this implication is speculative.

### Implications for educator preparation and intervention

If replicated, the findings would suggest that preparation for teachers of ELs at risk for MLD should attend to both cognitive and motivational aspects of word-problem solving. Because the present evidence is correlational and does not evaluate any instructional practice, the following are offered as hypotheses for intervention research rather than as recommendations for practice. Supports that reduce WM and language demands, such as explicit instruction in mathematical vocabulary, visual and schematic representations, and structured problem-solving routines, have been examined in intervention studies involving students with mathematics difficulty or learning disabilities (Driver and Powell, 2017; Powell et al., 2022). Whether such supports are especially beneficial for students with lower WM is an aptitude-by-treatment question that the present data cannot answer.

Similarly, motivational supports framed around general mathematics beliefs, value, and engagement, such as meaningful and culturally relevant problem contexts, opportunities for early success, and collaborative problem solving, are plausible components of multicomponent interventions (Eccles and Wigfield, 2020; Schukajlow et al., 2023), but their effects on word-problem accuracy for ELs at risk for MLD were not examined here. Experimental studies that cross cognitive and motivational supports would be needed to test whether their benefits are additive.

### Limitations

Several limitations should be considered when interpreting these findings. The analytic sample was relatively small, and missing data reduced the number of participants in some analyses, potentially lowering estimate precision. The sample was limited to third-grade Latino English learners (ELs) from one high-poverty urban district, with slightly more than half identified as at risk for mathematics learning disabilities (MLD). Thus, the findings may not generalize to students from other linguistic, socioeconomic, geographic, or instructional contexts. In addition, the analyses were secondary to an intervention study, and risk status overlapped with the intervention condition, limiting the ability to isolate MLD risk as an independent factor.

Measurement and design limitations also warrant caution. The working memory (WM) measures showed weak intercorrelations, poor fit for a one-factor model, and low internal consistency, so the CFWM score should be interpreted as a broad aggregate rather than as a distinct WM construct. Because the WM tasks were administered in English, phonological-loop scores may also have reflected English-language knowledge. The motivation measure included posttest data, showed a weak pretest–posttest correlation, and has limited validity evidence for EL populations. In addition, the indirect-effect and interaction analyses were underpowered and should not be interpreted causally. Although multiple-testing corrections were applied, the analyses were not preregistered, so exploratory findings should be viewed as hypothesis-generating. Finally, the study included only 12 classrooms; the sources of missing data could not be fully determined, and no fluid-reasoning exclusion criterion was applied.

### Future directions

Future research should use longitudinal designs with multiple measurement occasions to examine reciprocal relations among WM, mathematics motivation, and growth in word-problem solving. Larger, more diverse samples of ELs are needed to test whether the pattern of distinct associations generalizes across language backgrounds, grade levels, and instructional contexts. Studies that administer the mathematics motivation measure alongside measures of self-efficacy, expectancy-value beliefs, mathematics anxiety, and implicit theories of intelligence would help distinguish general mathematics motivation from more specific motivational constructs. Studies should also include tasks designed to tap the episodic buffer and administer WM tasks in both of students’ languages. Finally, randomized trials of multicomponent interventions could test whether combining cognitive scaffolds with motivational supports yields additive benefits for ELs at risk for MLD.

## Conclusion

This study offers cautious, correlational evidence that WM and general mathematics motivation are distinct correlates of posttest word-problem-solving accuracy among third-grade Latino ELs, with and without MLD risk, in a high-poverty urban district. Both associations were significant in the primary model and after correction for the confirmatory tests, but each was attenuated under some alternative specifications. Exploratory analyses did not detect mediation or moderation, although they were underpowered, and did not establish a distinct role for phonological WM. The findings are consistent with an integrated cognitive-motivational perspective on ELs’ word-problem solving and motivate longitudinal and experimental studies that test whether reducing linguistic and WM demands while supporting students’ engagement improves outcomes for ELs at risk for MLD.

## Data Availability

The datasets presented in this article are not readily available because the datasets generated during this study cannot be made available due to ethical restrictions protecting vulnerable study participants. Requests to access the datasets should be directed to Michael J. Orosco, mjorosco@ku.edu.
