# Supplementary material for: Working memory, mathematics motivation, and problem solving in English learners with and without risk for mathematics learning difficulties: evidence from a high-poverty urban district
Source: Front Psychol. 2026 Oct 19;17:1928492. doi: 10.3389/fpsyg.2026.1928492 (PMC13724579; doi:10.3389/fpsyg.2026.1928492)
Supplement: Supplementary file 1 [file Data_Sheet_1.PDF]

Supplementary Material

Supplementary Table S1

Content of the Mathematics Motivation Items

Item	Content (paraphrased)	Factor (two-factor model)	Loading	Pretest p	Posttest p
1	Enjoyment of doing mathematics	Beliefs/attitudes	.94	.77	.90
2	Positive vs. negative feelings (happy vs. sad) when doing mathematics	Beliefs/attitudes	.89	.81	.92
3	Feeling good vs. bad when doing mathematics work	Beliefs/attitudes	.89	.79	.89
4	Willingness to spend energy on mathematics	Engagement	.75	.64	.74
5	Preferring mathematics over other activities	Engagement	.84	.48	.65
6	Perceived ease vs. difficulty of mathematics	Beliefs/attitudes	.44	.79	.82
7	Perceived pace of mathematics work (goes fast vs. takes too long)	Beliefs/attitudes	.75	.65	.78
8	Excitement vs. boredom when doing mathematics	Engagement	.90	.62	.82
9	Importance of doing well in mathematics	Beliefs/attitudes	.78	.79	.94
10	Choosing to do mathematics because of liking it	Beliefs/attitudes	.87	.77	.85

Note. Each item presents a positive and a negative statement; students mark the statement that best describes them (positive = 1, negative = 0). Content descriptions are paraphrased; the full item wording is published in Appendix A of Author (2016). Factor assignments (two-factor model) and standardized loadings (one-factor model) are from the validation study (Author, 2016; N = 182 native English-dominant second and third graders). In that study, Item 6 had low discrimination (adjusted point-biserial r = .29), and Items 9 and 10 showed differential item functioning by gender. Pretest and posttest p = proportion of students in the present sample endorsing the positive statement (pretest n = 47–48; posttest n = 71–72).

Supplementary Table S2

Focal Coefficient Tests Across Confirmatory, Exploratory, and Sensitivity Models

Model and coefficient	*n*	*b*	*SE*	*p*	Adjusted
**Confirmatory family (Holm-adjusted *p*)**					
Model 2: WM	62	0.33	0.09	< .001	.002
Model 3: WM	62	0.28	0.09	.004	.008
Model 3: GMM	62	0.22	0.09	.019	.019
**Exploratory and sensitivity family (Benjamini–Hochberg *q*)**					
Model 4 (+ fluid reasoning, reading comprehension): WM	60	0.18	0.10	.071	.101
Model 4: GMM	60	0.20	0.10	.040	.076
Model 3 + intervention condition: WM	62	0.28	0.09	.005	.034
Model 3 + intervention condition: GMM	62	0.24	0.09	.014	.048
Model 3 + pretest WPS: WM	62	0.11	0.09	.211	.266
Model 3 + pretest WPS: GMM	62	0.16	0.08	.047	.084
Pretest-only GMM: WM	40	0.24	0.13	.059	.093
Pretest-only GMM: GMM	40	0.22	0.19	.234	.284
Posttest-only GMM: WM	62	0.32	0.09	< .001	.007
Posttest-only GMM: GMM	62	0.16	0.09	.060	.093
Model 4, cluster-robust SEs: WM	60	0.18	0.09	.032	.064
Model 4, cluster-robust SEs: GMM	60	0.20	0.14	.143	.187
Model 3 + English proficiency: WM	57	0.23	0.10	.026	.055
Model 3 + English proficiency: GMM	57	0.25	0.09	.006	.034
Model 3, multiple imputation: WM	76	0.30	0.08	< .001	.007
Model 3, multiple imputation: GMM	76	0.20	0.08	.022	.054
Model 4, multiple imputation: WM	76	0.23	0.09	.011	.042
Model 4, multiple imputation: GMM	76	0.14	0.08	.105	.143
Model 3, unit-weighted WM: WM	72	0.31	0.09	< .001	.007
Model 3, unit-weighted WM: GMM	72	0.24	0.09	.009	.038
Model 4, unit-weighted WM: WM	69	0.23	0.09	.007	.034
Model 4, unit-weighted WM: GMM	69	0.17	0.09	.070	.101
Oral composite (CMAT, KeyMath-3): WM	62	0.31	0.10	.003	.026
Oral composite (CMAT, KeyMath-3): GMM	62	0.24	0.10	.023	.054
TOMA-2 (reading-dependent): WM	62	0.24	0.10	.019	.054
TOMA-2 (reading-dependent): GMM	62	0.24	0.10	.018	.054
Subcomponents: central executive	61	0.36	0.18	.058	.093
Subcomponents: phonological loop	61	0.29	0.13	.024	.054
Subcomponents: visuospatial sketchpad	61	−0.02	0.12	.854	.880
Phonological loop − central executive	72	−0.04	0.26	.884	.884
Path a: WM → GMM	63	0.09	0.14	.533	.585
WM × GMM interaction	62	0.03	0.10	.803	.853
Risk × WM	62	0.13	0.18	.460	.521

Model and coefficient	*n*	*b*	*SE*	*p*	Adjusted
Risk × GMM	62	−0.17	0.20	.401	.470

Note. WM = working memory (CFWM unless otherwise indicated); GMM = general mathematics motivation (pretest–posttest average unless otherwise indicated); WPS = word-problem solving. Adjusted values are Holm-adjusted *p* values for the confirmatory family (3 tests) and Benjamini–Hochberg *q* values for the exploratory family (34 tests). Models from the original submission were estimated with SAS PROC MIXED. Models added in revision were estimated as REML multilevel models, except the multiple-imputation models ($m = 50$), which were single-level regressions; the phonological loop − central executive contrast was estimated with PROC MIXED. Subcomponent estimates are from the model with all three components entered simultaneously. Estimates involving intervention condition or risk status are provisional.
